# The Elusive Third Subunit IIa of the Bacterial B-Type Oxidases: The Enzyme from the Hyperthermophile *Aquifex aeolicus*

**DOI:** 10.1371/journal.pone.0021616

**Published:** 2011-06-30

**Authors:** Laurence Prunetti, Myriam Brugna, Régine Lebrun, Marie-Thérèse Giudici-Orticoni, Marianne Guiral

**Affiliations:** 1 Laboratoire de Bioénergétique et Ingénierie des Protéines, UPR 9036, Institut de Microbiologie de la Méditerranée (IFR88)-Centre National de la Recherche Scientifique, Marseille, France; 2 Université de Provence, Marseille, France; 3 Plate-forme Protéomique de l'IFR88-Centre National de la Recherche Scientifique, Marseille Protéomique, Marseille, France; National Institute for Medical Research, Medical Research Council, London, United Kingdom

## Abstract

The reduction of molecular oxygen to water is catalyzed by complicated membrane-bound metallo-enzymes containing variable numbers of subunits, called cytochrome *c* oxidases or quinol oxidases. We previously described the cytochrome *c* oxidase II from the hyperthermophilic bacterium *Aquifex aeolicus* as a *ba*_3_-type two-subunit (subunits I and II) enzyme and showed that it is included in a supercomplex involved in the sulfide-oxygen respiration pathway. It belongs to the B-family of the heme-copper oxidases, enzymes that are far less studied than the ones from family A. Here, we describe the presence in this enzyme of an additional transmembrane helix “subunit IIa”, which is composed of 41 amino acid residues with a measured molecular mass of 5105 Da. Moreover, we show that subunit II, as expected, is in fact longer than the originally annotated protein (from the genome) and contains a transmembrane domain. Using *Aquifex aeolicus* genomic sequence analyses, N-terminal sequencing, peptide mass fingerprinting and mass spectrometry analysis on entire subunits, we conclude that the B-type enzyme from this bacterium is a three-subunit complex. It is composed of subunit I (encoded by *coxA_2_*) of 59000 Da, subunit II (encoded by *coxB_2_*) of 16700 Da and subunit IIa which contain 12, 1 and 1 transmembrane helices respectively. A structural model indicates that the structural organization of the complex strongly resembles that of the *ba*_3_ cytochrome *c* oxidase from the bacterium *Thermus thermophilus*, the IIa helical subunit being structurally the lacking N-terminal transmembrane helix of subunit II present in the A-type oxidases. Analysis of the genomic context of genes encoding oxidases indicates that this third subunit is present in many of the bacterial oxidases from B-family, enzymes that have been described as two-subunit complexes.

## Introduction

In aerobic respiration of prokaryotic and eukaryotic organisms, the reduction of molecular oxygen to water is catalyzed by terminal oxidases which are integral membrane multi-subunit enzymatic complexes belonging to the heme-copper oxidases superfamily [1]. These respiratory enzymes, which also pump protons across membrane, have been named cytochrome *c* oxidases or quinol oxidases, depending on the electron donor that they oxidize. A classification of these enzymes into three families was proposed by Pereira *et al.* [2]: i) type A (mitochondrial-like oxidases), ii) type B (*ba*_3_-type oxidases) and iii) type C (*cbb*_3_-type oxidases). More recently, Hemp and Gennis identified five novel oxygen reductase families found exclusively in Archaea [3]. The subunit composition of types A and B enzymes differs from one oxidase to another but these heme-copper oxidases always contain the catalytic subunit (subunit I) and a smaller subunit named subunit II. Subunit I is an integral membrane protein composed of at least 12 transmembrane helices, containing the heme-copper binuclear centre Cu_B_ and its immediate electron donor, a low-spin heme. Subunit II of oxidases A and B is made of a transmembrane domain and a peripheral one in which the copper Cu_A_ centre is located. This subunit binds the electron donor (reduced cytochrome *c*, but also other metalloproteins) and transfers electrons to subunit I [2].

*Aquifex aeolicus* (*A. aeolicus*) belongs to the *Aquificales* order of Bacteria and is a hyperthermophilic chemolithoautotrophic microorganism using molecular hydrogen as electron donor and molecular oxygen as electron acceptor in the presence of a sulfur compound. The *Aquifex* genome sequence analysis revealed the presence of genes coding for enzymes potentially involved in oxygen reduction: two cytochrome *c* oxidases and a cytochrome *bd* quinol oxidase [4], [5]. Based on sequence comparison, Pereira *et al.* proposed that cytochrome *c* oxidase I from *A. aeolicus* (putatively encoded by *coxA_1_*, *coxB* and *coxC*) belongs to type A and has all the specific residues of the D- and K- protons channels, whereas cytochrome *c* oxidase II (encoded by *coxA_2_* and *coxB_2_*) is a B-type enzyme with specific residues forming a K-channel homologue for proton translocation [2]. Our previous work on *A. aeolicus* membranes showed the presence of the *bd* quinol oxidase and the cytochrome *c* oxidase II when the bacterium grows with molecular hydrogen, molecular oxygen and elemental sulfur [5]. We have recently described a new multienzyme supercomplex carrying a sulfide oxidase-oxygen reductase activity that contains the sulfide quinone reductase (Sqr), the dimeric *bc*_1_ complex and the cytochrome *c* oxidase II [6]. We demonstrated that this latter enzyme is a *ba*_3_-type oxidase and that it is composed of at least the catalytic subunit I and the small subunit II [6], in agreement with its classification in the B family, as proposed by Pereira [2]. However, a novel small subunit belonging to the type B *ba*_3_ cytochrome *c* oxidase from *Thermus (T.) thermophilus*, called subunit IIa, was discovered in 2000 with the resolution of the crystal structure of this complex, implying that this B-type enzyme is thus a three-subunit oxidase [7], [8]. This novel subunit has 34 amino acid residues and forms a single helix across the membrane, corresponding in space to the first transmembrane helix of subunit II of the A-type cytochrome *c* oxidases [2], [7], [8].

Concerning the *ba*_3_ cytochrome *c* oxidase from *A. aeolicus*, there were some points that were intriguing to us: on the one hand the very small theoretical molecular mass of subunit II (about 9000 Da), and on the other hand the lack of a transmembrane segment for this protein as found in the NCBI database (accession NP_214505.1). Moreover, the presence of a third subunit in the *T. thermophilus* enzyme prompted us to characterize in more detail the subunit composition of the *ba*_3_ cytochrome *c* oxidase from *A. aeolicus*. We now demonstrate the existence of a third small helical subunit IIa in the oxidase complex belonging to the B family in *A. aeolicus*. This enzyme is thus only the second in which this subunit has been biochemically evidenced. Furthermore, we propose that the presence of this small protein is common in type B of oxidases, at least in the bacterial organisms.

## Results

### *A. aeolicus* genomic sequence analysis

#### Subunit II: detection of an error in the genomic sequence

**Figure 1:**
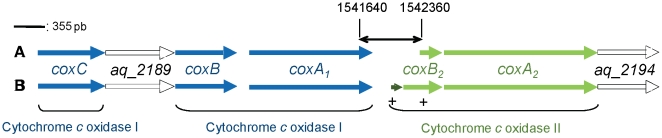
*A. aeolicus* oxidase gene cluster. Genes encoding the putative cytochrome *c* oxidase I (*coxA_1_*, *coxB* and c*oxC*) are shown in blue and genes encoding cytochrome *c* oxidase II (*coxA_2_* and *coxB_2_*) are shown in green. Genes encoding hypothetical proteins putatively involved in synthesis of cytochrome *c* oxidases (SCO family) are shown in white. A: ORFs derived from the annotation of the genomic sequence previously published [4]. B: ORFs found after a careful examination of the sequence spanning from nucleotides 1541640 (inside the *coxA_1_* sequence) to 1542360 (inside the *coxB_2_* sequence), symbolized by the double-headed arrow. Positions of oligonucleotides pairs used to generate DNA fragment for DNA sequencing are indicated with small crosses (+). *coxB_2_* is extended and a new ORF, encoding subunit IIa, is detected upstream *coxB_2_* gene (dark green arrow). The scale bar (upper left) represents 355 pb.

We previously showed that cytochrome *c* oxidase II, belonging to the family B of oxidases, is synthesized in the membranes of *A. aeolicus* in our growth conditions and that it is a *ba*_3_ enzyme [5], [6]. We decided to check the genomic deoxyribonucleic acid (DNA) sequence for a potential error of sequencing. As in the oxidase cluster grouping 7 genes, a relatively large non-coding nucleotide sequence stands between *coxA_1_* and *coxB_2_* genes (Figure 1, nucleotides 1541640 to 1542360), this region has been carefully analyzed (with potential open reading frame (ORFs) detection and sequence comparison). A probable insertion of one nucleotide at the position 1542190 caused a frame-shift. We confirmed this error in the genomic sequence by sequencing the *A. aeolicus* DNA located between oligonucleotide pairs (centred on positions 1541997 and 1542342, Figure 1). This led to the identification of a possible new start for the *coxB_2_* gene, coding for a putative longer protein (extended at its N terminal part with 64 amino acid residues) with a theoretical molecular mass of 16700 Da which is compatible with the experimental one (see next section). A BLAST search using the corrected protein sequence indicates high similarity with proteins, annotated *cytochrome c oxidase subunit II*, from bacteria belonging to *Aquificales* order (*Hydrogenivirga* sp., *Thermocrinis albus*, and *Hydrogenobacter thermophilus*, 74%, 61% and 56% identity respectively) (Supplementary Figure S1). Some similarities were also found with other bacterial and archaeal proteins, including the characterized *ba*_3_ cytochrome *c* oxidases from *T. thermophilus* (31% identity) [7], *Rhodothermus (R.) marinus* (36% identity) [9] and *Geobacillus* (*G.*) *stearothermophilus* (36% identity) [10], [11]. Sequence comparison of subunit II shows that: (1) length of *A. aeolicus* subunit II is in the same range of oxidases from the B-family, and (2) amino acid residues involved in the binuclear copper Cu_A_ centre coordination (His 96, Cys 131, Cys 135, His 139, Met 142) are conserved in the *A. aeolicus* sequence. Moreover, a prediction of secondary structures for the corrected subunit II sequence indicates the presence of only one transmembrane helix which constitutes the domain of insertion of the protein in the membrane as described for subunit II from B-type enzymes (instead of two helices found in the A-type oxidases) [2], [7], [8], [10], [12] (Figure S1). This transmembrane domain was previously not described because of the error in the genomic sequence [2].

#### Subunit IIa: detection of an additional subunit

**Figure 2:**
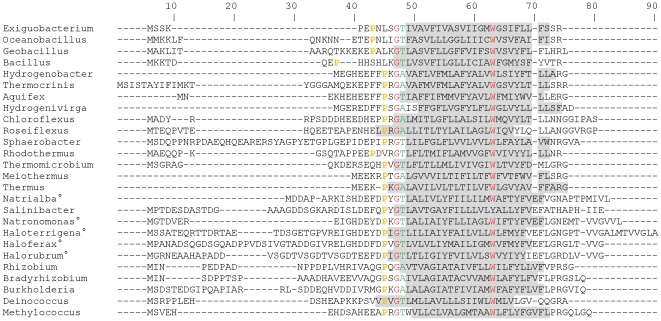
Multiple sequence alignment of putative bacterial and archaeal oxidase subunits IIa. These proteins correspond to putative or characterized *ba*_3_ cytochrome *c* oxidases. Residues identical in all sequences are shown in red (except the Tyr from the *Salinibacter* sequence), strongly similar residues in green. The Proline residue present in all sequences in the motif PX_1–3_GT/A is shown in orange. Grey shaded residues are involved in a putative transmembrane helix (Transmembrane region prediction with TMHMM program, http://www.cbs.dtu.dk/services/TMHMM-2.0/). Grey shaded residues in the *Thermus* sequence form a transmembrane helix in the three-dimensional structure of the enzyme (PDB 1XME). The alignment has been made with ClustalW. Complete names of organisms and accession numbers of sequences used to create the alignment are given in Table 1. The symbol ° indicates archaeal organisms.

The gene coding for the small subunit IIa (*cbaD*) of *T. thermophilus* is located directly upstream from that for the subunit II in the operon coding for the *ba*_3_ oxidase [8]. We thus decided to search for a potential third subunit gene that would be located upstream the one of subunit II, in the genome of *Aquifex*. Directly upstream of *coxB_2_* gene, we found a small ORF encoding a putative protein of 41 amino acid residues with a theoretical monoisotopic molecular mass of 5104.46 Da (Figure 1, nucleotides 1541953 to 1542075). A search with the BLAST program using this sequence indicates similarities with very few proteins: two proteins from the *Aquificales Hydrogenobacter thermophilus* and *Thermocrinis albus* and one annotated *cytochrome c oxidase subunit IIa* from the bacterium *Meiothermus silvanus* (in *cox* operon in the three cases). This potential small subunit is thus probably the third subunit, subunit IIa of the *ba*_3_ cytochrome oxidase. We compared, with a sequence alignment, the novel *A. aeolicus* sequence of subunit IIa with the one from subunit IIa from *T. thermophilus* (34 amino acid residues). This shows 60% of residues with similar chemical properties (including 30% identity) between the two peptides (Figure 2). The prediction indicates that this small subunit is probably a transmembrane helix. Taken together, all these results support the existence of a small helical third subunit in the *ba*_3_ cytochrome *c* oxidase from *A. aeolicus*.

### Experimental evidence for the *A. aeolicus* subunits II and IIa and structural model of the complex

As the *ba*_3_ cytochrome *c* oxidase from *A. aeolicus* cannot be separated by purification from its tightly bound partners (*bc*_1_ complex and sulfide quinone reductase), we worked with the entire supercomplex in search of evidence for the presence of the putative subunit IIa, and to confirm the new N-terminal sequence of the subunit II.

**Figure 3:**
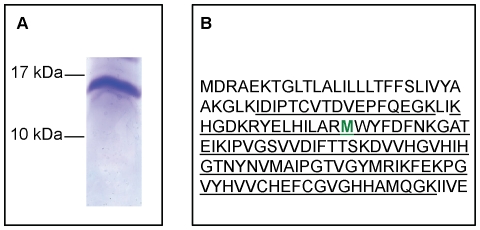
Identification of subunit II of the *A. aeolicus* oxidase after migration on a SDS gel. A: 70 µg of the entire supercomplex containing the sulfide quinone reductase, the *bc*_1_ complex and the cytochrome *c* oxidase II [6] was loaded on a 10–20% Tris-Tricine gel. The proteins were stained with Coomassie Blue. The molecular weight markers are indicated on the left. Only subunit II is shown. B: MALDI-TOF mass spectrometry analysis of subunit II (∼17 kDa band in A). Underlining indicates peptide sequences identified by mass spectrometry (75% sequence coverage). The bold green methionine residue corresponds to the erroneous N-terminal extremity of the subunit as determined after genome sequencing.

A separation of the supercomplex subunits on a 10–20% denaturing gel did not allow us to visualize a small protein around 5 kDa, as was also the case for the same subunit from *T. thermophilus* [8]. Subunit II migrated on this gel at a position corresponding to a protein of slightly less than 17 kDa (Figure 3 A), corresponding to the correct molecular mass. After transfer of the protein from this gel band, N-terminal sequencing of the first 12 amino acid residues allowed to confirm the N terminal sequence (MDRAEKTGLTLA-) of subunit II determined from the genomic sequence (shown in Figure 3 B). In addition, a second protein was clearly detected in the same sample with a distinct N-terminal sequence, except for the first Met, (MNEKHXXXEF-, X being an undetermined residue) matching the N-terminal part of the proposed subunit IIa (Figure 2). This corroborates the existence of this additional protein in the *A. aeolicus* oxidase and shows the strong interaction between subunits II and IIa as they are not dissociated after migration on a sodium dodecyl sulfate (SDS) gel. The unexpected migration of the complex subunit II-subunit IIa at this size might be explained by the hydrophobic nature of the proteins, as previously pointed out [13]. The analysis of the protein band at 17 kDa by peptide mass fingerprinting after trypsin digestion showed assignment of ionic peptides spanning more than 75% of the subunit II sequence, including some in the extended N-terminal part (upstream from the previously proposed N-terminal) (Figure 3 B), whereas ions from subunit IIa were not detected, probably because of the highly hydrophobic nature and the very small size of this protein.

**Figure 4:**
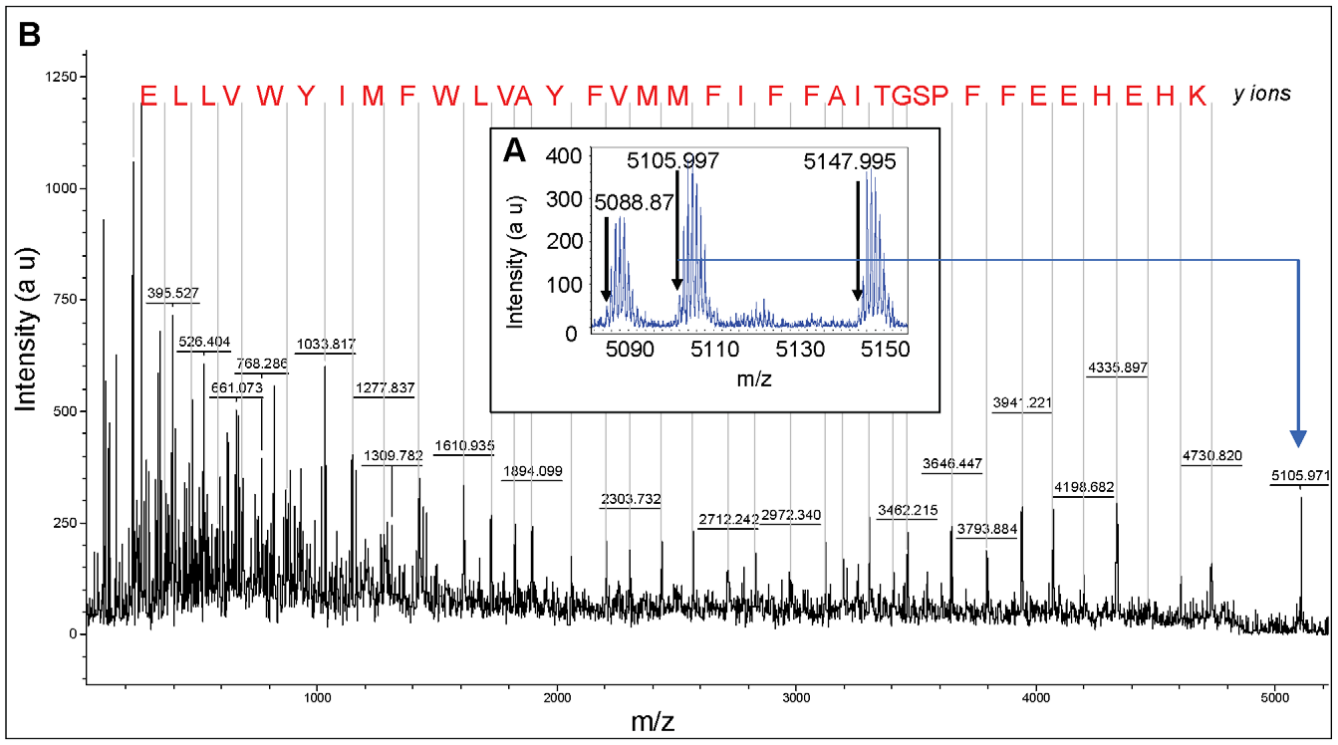
MALDI-TOF MS and MS/MS of subunit IIa. A: Three monoisotopic distributions have been distinguished in reflecton and positive mode (range 5000–5200): m/z 5105.99 assigned to the subunit IIa (calculated m/z 5105.47, 103 ppm), m/z 5088.87 and m/z 5147.99, at −17 Da and +42 Da respectively, compared to m/z 5105.99. B: The ion precursor at m/z 5105.99 was fragmented in a lift mode, and a continuous series of *y* ions, manually assigned (rms = 0.5 Da), could elucidate the sequence of subunit IIa from Lys 4 to Glu 39.

The molecular masses of both subunit II and subunit IIa were determined by Matrix-Assisted Laser Desorption/Ionization-Time-of-flight (MALDI-TOF) mass spectrometry using the intact supercomplex solution. As expected, the mass spectrum revealed several species corresponding to the constituents of the supercomplex (data not shown). In reflectron and positive mode, an ion has been measured at m/z 5105.99 and could be assigned to the subunit IIa (theoretical m/z 5105.47, Δm = 103 ppm). None of the other supercomplex components has a molecular mass that can be assigned to this latter one. Two other masses are also observed: one at m/z 5088.87, a 17 Da lower mass which could correspond to this subunit without one -NH_3_ group, the other observed at m/z 5147.99, a 42 Da heavier mass which could correspond to an addition of an acetyl group (Figure 4 A). The ion precursor at m/z 5105.99 was enough intense to be fragmented in a lift mode, and a continuous series of *y* ions could unambiguously elucidate the sequence of subunit IIa from Lys 4 to Glu 39 (Figure 4 B). In linear mode, an intense ion was observed at m/z 16725.2 (data not shown) and could probably correspond to the subunit II (theoretical m/z 16701.5), with an addition of one Na^+^ (m/z 16724.4, Δm = 48 ppm).

These experiments confirmed that subunit II is longer than previously annotated and that a third subunit is present in the *ba*_3_ oxidase from *A. aeolicus*.

**Figure 5:**
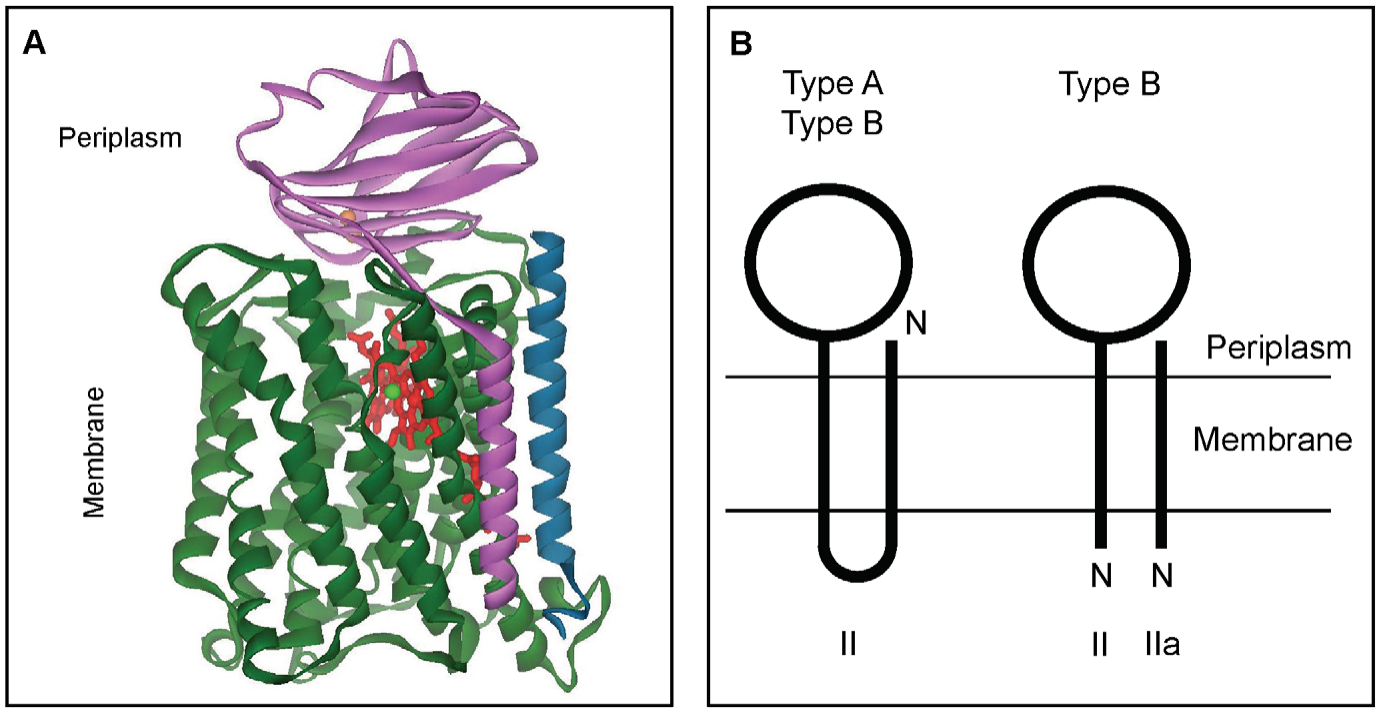
Structural organization of *A. aeolicus ba*_3_ oxidase and of subunits II/IIa in A and B-type oxidases. A : Ribbon display of the overall modeled structure calculated with SWISS-MODEL using the crystal structure of the *ba*_3_ oxidase from *T. thermophilus* (PDB entry 1XME) as a template (See Materials and methods for details). View parallel to the membrane showing subunit I (CoxA_2_) in green, subunit II (CoxB_2_) in pink, subunit IIa in blue, and the cofactors (hemes A and B in red, copper ion Cu_B_ in green and the binuclear copper Cu_A_ site in orange). This figure was made with WebLab ViewerLite 5.0 (Accelrys). B : Schematic representation of the topology of the membrane-spanning helices of subunits II and IIa in the two types A and B of terminal oxidases [8]. N indicates the N-terminal extremity of the subunits, II and IIa indicate subunit II and subunit IIa respectively. The transmembrane and the periplasmic domains are shown.

A model of the three-dimensional structure of *A. aeolicus ba*_3_ cytochrome oxidase has been calculated with SWISS-MODEL protein modeling program using the structure of the *ba*_3_ cytochrome oxidase from *T. thermophilus* as a template (PDB entry 1XME) [14]. An overall view of the structure is presented in Figure 5 A. The complex is composed of three protein subunits, I, II and IIa.

Whereas *T. thermophilus ba*_3_ oxidase subunit I is composed of 13 transmembrane-spanning helices, *Aquifex* subunit I consists of only 12 transmembrane helices. Heme B and the binuclear centre heme [A_3_.Cu_B_] reside in subunit I. Hemes and copper ions were included in the structural model from the crystal structure of the *ba*_3_ oxidase from *T. thermophilus* in which the A-type heme corresponds to the heme A_s_. The heme A_s_, first isolated in the SoxB-type terminal oxidase from the thermoacidophilic archae *Sulfolobus acidocaldarius*, is a A-type heme in which the farnesyl chain is replaced by a geranylgeranyl group [15]. Unlike *T. thermophilus*, *A. aeolicus* seems to contain heme A in the membrane, as shown for *Aquifex pyrophilus* [15]. Therefore, the heme A_s_ has been modified to a heme A in the structural model of the *A. aeolicus ba*_3_ oxidase (Figure 5 A). The two axial ligands of heme B are the two conserved histidine residues His 60 and His 385. The histidine residue His 383 is the axial ligand of the heme A_3_ and the three histidine ligands to Cu_B_ are His 222, His 273 and His 274.

Subunit II has only one transmembrane helix which is in close contact with subunit I. The periplasmically oriented polar domain of subunit II contains the binuclear copper Cu_A_ site. The Cu_A_ site is symmetrical with respect to the two histidine (His 96 and His 139) and the two cysteine (Cys 131 and Cys 135) ligands (Figure S1).

The third subunit of the complex, subunit IIa, consisting of 41 amino acid residues, forms one transmembrane helix. This helix, also found in the structure of the *ba*_3_ oxidase from *T. thermophilus*, is superimposable with the first transmembrane helix of subunit II from the A-type *aa*_3_ cytochrome oxidase from *Paracoccus denitrificans*, but with opposite polarity [8] (Figure 5 A and B).

### Presence of the subunit IIa in B-type cytochrome c/quinol oxidases

To determine whether subunit IIa is present in the majority of enzymes in the B family, or on the contrary, whether it is rare, we searched for a gene potentially encoding this subunit IIa in the genomes of various micro-organisms. We have investigated the existence of the peptide IIa, at the genomic level, in (1) B-type putative prokaryotic oxidases [12], [16], [17], [18], and in (2) biochemically characterized *ba*_3_ oxidases (*R. marinus*, *Geobacillus* sp. and *Natronomonas pharaonis*) [9], [10], [11], [19], [20]. In each case, we retrieved the sequence of the gene located directly upstream of the subunit II gene of the oxidase. When present, it is annotated as *hypothetical protein* in most cases, apart from the one in *Meiothermus*, which is annotated *cytochrome c oxidase subunit IIa*, those in *Methylococcus*, *Natronomonas* and *Haloferax*, annotated *cbaD subunit*, and the one found in *Natrialba*, annotated *halocyanin*. When such a gene coding for a putative peptide IIa was not present, we directly analyzed the DNA upstream the subunit II gene to find a potential small ORF, possibly not detected at the time of automatic genome annotation. We analyzed 36 oxidase genes region, found 21 putative subunit IIa sequences and detected 6 previously unannotated ORFs (Table 1). Sequence alignment confirms that the closest sequences to the *A. aeolicus* sequence belong to organisms in the *Aquificales* order such as *Hydrogenivirga*, *Thermocrinis* and *Hydrogenobacter* (Figure 2). It seems that archaeal subunits (53 to 78 amino acid residues) are longer than the bacterial ones (34 to 71 residues). Although sequence similarities between archaeal and bacterial organisms are low, two residues are conserved in all the 27 sequences (Gly 14 and Trp 30, *A. aeolicus* numbering), except that Glycine is absent in the sequence of *Magnetospirillum* and Tryptophan is replaced by Tyrosine in that of *Salinibacter*. In addition, the motif PX_1–3_GT/A (Pro 12, *A. aeolicus* numbering) appears to be present in most cases (Figure 2). It seems that this sequence motif is absent from subunit II from A and B-type oxidases possessing two transmembrane helices. The role of this recurrent sequence pattern is unknown.

**Table 1:** Properties of characterized or putative subunit II and subunit IIa from some bacterial and archaeal oxidases belonging to the B-family.

		Subunit II			Subunit IIa	
Organism	Accession	TM helix	MM	Accession	TM helix	MM
*Aquifex aeolicus*	NP_214505	1	16700	Not annotated	1	5108
*Thermus thermophilus*	YP_144400	1	18563	YP_144399	1	3766
*Exiguobacterium siribium*	YP_001813095	1	13809	YP_001813094	1	3825
*Thermomicrobium roseum*	YP_002521803	1	16597	YP_002521802	1	4919
*Bacillus halodurans*	NP_241606	1	17447	NP_241607	1	4638
*Rhodothermus marinus*	YP_003290840.1	1	17263	YP_003290841.1	1	5371
*Roseiflexus castenholzii* DSM 13941	YP_001431685	1	18069	YP_001431684	1	6065
*Methylococcus capsulatus str. Bath*	YP_114812	1	19354	YP_114814	1	5354
*Burkholderia multivorans* ATCC17616	YP_001585718	1	21154	YP_001585719	1	7148
*Oceanobacillus iheyensis* HTE831	NP_692666	1	18390	Not annotated	1	5071
*Chloroflexus aurantiacus* J-10-fl	YP_001636023	1	13236	YP_001636022	1	5824
*Bradhyrizobium japonicum* USDA 110	NP_771121	1	19992	Not annotated	1	5972
*Thermocrinis albus* DSM 14484	YP_003474329	1	16155	YP_003474328	1	6366
*Geobacillus spC56T3*	YP_003671527	1	16881	YP_003671526	1	5387
*Hydrogenivirga* sp.128-5-R1-1	ZP_02176551	1	16560	ZP_02176552	1	4355
*Hydrogenobacter thermophilus* TK-6	YP_003433136	1	16543	YP_003433135	1	4255
*Sphaerobacter thermophilus* DSM 20745	YP_003320090	1	13114	YP_003320089	1	7847
*Meiothermus silvanus* DSM 9946	YP_003685401	1	17733	YP_003685402	1	3944
*Salinibacter ruber* DSM 13855	YP_444459	1	18901	Not annotated	1	7196
*Deinococcus geothermalis* DSM 11300	YP_603489	1	19744	Not annotated	1	5529
*Magnetospirillum magneticum* AMB-1	YP_421584	1	20462	YP_421583	1	5828
*Rhizobium etli* CFN42	YP_468521	1	19731	Not annotated	1	5911
*Natrialba magadii* ATCC 43099*°*	YP_003481905	1	28596	YP_003481904	1	6080
*Haloferax volcanii* DS2°	YP_003535001	1	19378	YP_003535000	1	7986
*Natronomonas pharaonis°*	CAA71530	1	18628	CAA71529	1	6081
*Haloterrigena turkmenica* DSM 5511*°*	YP_003402035	1	27982	YP_003402036	1	8496
*Halorubrum lacusprofundi* ATCC 49239°	YP_002567057	1	15188	YP_002567056	1	7223
*Nitrobacter hamburgensis* X14	YP_577951	2	19699	Absent	-	-
*Silicibacter pomeroyi* DSS-3	YP_165014	2	18559	Absent	-	-
*Geobacillus kaustophilus* HTA426	YP_147523.1	2	20134	Absent	-	-
*Sulfurimonas denitrificans* DSM 1251	YP_392619	2	22228	Absent	-	-
*Nitrosomonas europaea* ATCC 19718	NP_840763	2	19364	Absent	-	-
*Nocardioides* sp. JS614	YP_922822	2	21603	Absent	-	-
*Nitrosococcus oceani* ATCC 19707	YP_344943	2	19857	Absent	-	-
*Aromatoleum aromaticum* EbN1	YP_159093	2	19656	Absent	-	-
*Sulfurovum sp.* NBC37-1	YP_001357603	2	23477	Absent	-	-

Sequences were retrieved and analyzed as detailed in the Materials and Methods section. Putative subunits IIa without accession numbers (*Not annotated*) were detected in this work. Accession: NCBI accession number; TM helix: predicted transmembrane helices; MM: theoretical molecular mass in Da. The symbol ° indicates archaeal organisms.

Secondary structure prediction indicates that all these subunit IIa sequences contain one transmembrane helix, like that in *T. thermophilus* (Figure 2 and Table 1). Our analysis suggests that the helical peptide is present in many (75%) of the oxidase complexes studied. All these enzymes possess a one-helix transmembrane domain in subunit II. However, this subunit IIa was not detected when subunit II is predicted to have two membrane-spanning helices (small number of analyzed oxidases [12], Table 1). This confirms the idea that this transmembrane helix IIa is structurally equivalent to the missing helix of subunit II [7], [8]. Although we did not analyze all the B-type cytochrome oxidases available in the genomic data, it seems that this third subunit IIa is widespread in the bacterial *ba*_3_-type enzymes.

All operons analyzed in this study have the same genomic organization as found for *Thermus* [8] and *Aquifex ba*_3_ oxidases, with at least three genes encoding the oxidase, for the large subunit I, for the small subunit II and for a third subunit IIa (Figure 1). An exception is the enzyme from *Methylococcus capsulatus*, where one gene (coding for a hypothetical small protein) is located between the genes encoding the putative subunit IIa and the subunit II. It should be noted that archaeal oxidases seem to contain at least one more subunit than the bacterial B-type enzymes, as illustrated by the purified oxidase from *N. pharaonis* [19], [20].

## Discussion

Cytochrome *c* oxidases are essential enzymes in the respiration of aerobic organisms. The A-family of heme-copper oxidases is the largest and the best studied, whereas few type B enzymes have been purified and fully characterized. We previously proposed that the cytochrome *c* oxidase II from *A. aeolicus* (B-family) is a *ba*_3_-type enzyme [6]. We show in this work that it is in fact composed of three subunits, subunit I, subunit II and subunit IIa. The name *coxIIa* can be attributed to the new gene coding for the subunit IIa. We moreover propose that the small hydrophobic subunit IIa is present in a large number of oxidases in the B family.

*Aquifex* is the second bacterium, after *T. thermophilus*, for which the existence of a third subunit in this type of oxidase has been experimentally demonstrated. Indeed, a *ba*_3_ oxidase was purified from *R. marinus* and from *G. stearothermophilus* and described in both cases as two-subunit enzymes, although we show here that *coxIIa* gene is present in the genome of *Rhodothermus* and *Geobacillus* sp. C56T3 (this strain is homologous to *G. stearothermophilus*, with more than 93% identity between subunits II of the B-type oxidases from both organisms) (Figure 2 and Table 1). In Archaea, although subunits I and II were detected in the *ba*_3_ oxidase from *Aeropyrum pernix* [21], the enzyme from *N. pharaonis* contains in addition a smaller subunit that is lost during purification [19], [20]. This underlines the difficulty of detecting subunit IIa in these complexes at the protein level, probably because of its high hydrophobic nature and its very small size. Indeed, it is not visible on a denaturing gel and is not amenable to identification by MALDI TOF after trypsin digestion. Moreover, the fact that the gene encoding subunit IIa is not always annotated in genomes makes it hard to detect at the genomic level.

Our analysis of the genetic environment of some oxidase subunit II genes suggests that subunit IIa is a general feature of the bacterial B-type oxidases that possess only one transmembrane spanning helix. These enzymes are thus three-protein complexes, subunits IIa and II being, as previously postulated, the structural equivalents of subunit II of type A enzymes [7], [8], [22]. A gene fission, located between the two transmembrane helices, of the gene coding for the two-helices subunit II or in contrary a fusion of the genes coding for the two subunits II and IIa (Figure 5 B) might have occurred during evolution of the oxidases. A systematic and comprehensive study proposed that there is a correlation of gene fission with thermophily and that split genes might reflect an adaptation to high temperature [23]. However, our present study points that subunit IIa is potentially present in organisms that are phylogenetically diverse and that this presence is not limited to thermo or hyperthermophilic species (Table 1). This study shows also that some bacterial oxidases from family B are inserted in the membrane by two transmembrane helices, like the type A enzymes (Table 1), as previously proposed in [12]. In these cases, no subunit IIa has been found. In Archaea, type B oxidases appear to be more diverse in terms of composition, size and nature of subunits than in Bacteria. A recent study, based on sequences analysis, proposes a reclassification of the archaeal B-type heme-copper oxidases in new families [3]. According to these authors, subunit IIa seems to be present in archaeal oxidases of only one family of this type of enzymes [3]. It is noteworthy that this small protein IIa occurs as a fusion protein with halocyanin in Haloarchaea, the blue copper halocyanin being described as the redox partner of the *ba*_3_ cytochrome oxidase in *N. pharaonis* [3], [20], [24]. Indeed, in *Halobacterium* and *Haloarcula*, the equivalent of subunit IIa is found at the C-terminal extremity of a protein annotated *halocyanin* (which gene is directly upstream the one for subunit II), the motif ProXaaGlyThr (Xaa is an unspecified amino acid) and the Trp residue (Figure 2) being conserved (Figure S2). Thus in prokaryotes, the membrane-spanning helix IIa appears to be present in a large number of oxidases either as an independent subunit (most of B-type oxidases, Table 1), or as a protein fusion with subunit II of oxidases (A-type oxidases and some B-type oxidases as in Table 1) or as a protein fusion with halocyanin (B-type enzymes in some Haloarchaea). This is reminiscent of the case of the “long” versus “split” cytochrome *b* from *bc* complexes. The majority of cytochromes *b* in Bacteria consists of a common “long” protein of eight membrane-spanning helices, whereas cyanobacteria and Firmicutes contain two separate proteins, cytochrome *b*_6_ and SUIV, corresponding to the N and C-terminal parts of the “long” cytochrome *b* respectively [25]. Moreover, in a few Firmicutes, SUIV is fused to a *c*-type cytochrome subunit [25]. Our preliminary analyses seem to indicate that there is no sequence similarity between subunit IIa and the transmembrane segment of the B and A-type oxidases (data not shown). A more in-depth investigation needs to be carried out to determine the evolutionary history of the subunit IIa of the type B oxidase complexes in Bacteria and Archaea.

Over-expression of only the two subunits I and II of the *ba*_3_-type oxidase from *G. stearothermophilus* led to an enzymatically active enzyme with proton pumping activity [11], which suggests that subunit IIa may not to be essential for the *ba*_3_ cytochrome oxidase activity. Nevertheless, as it is a homologous expression and in view of the difficulty to detect subunit IIa in the complex, we cannot absolutely rule out the possibility that a fraction of the purified oxidase could contain subunit IIa. The fact that the presence of two helices (separate or fused to another protein) in the transmembrane region of subunit II of heme-copper oxidases, a well preserved feature through evolution, suggests that it contributes to the stability, positioning, or interaction of the various subunits within the complex or of the whole complex in the membrane. Small subunits, predicted to form a single transmembrane helix, have been described in eukaryotic membrane protein respiratory or photosynthetic complexes [26]–[30]. In most cases, they are proposed to have a function in assembly, stabilization or dimerization of the complexes. Such a subunit can be found also in the bacterial *bc*_1_ complex of *Rhodovulum sulfidophilum* where it seems to play a stabilizing role [31]. Two additional small subunits containing a single transmembrane helix each were recently identified in the cyanobacterial NADH dehydrogenase complex [32].

Phylogenomic analysis highlighted the high level of redundancy of oxidase in prokaryotes [3], [16], [18]. As shown by biochemistry and bioinformatics, numerous prokaryotes possess both *aa*_3_- and *ba*_3_-type oxidases [12], [17], [18], [21], [33], [34]. Based on genome analysis, *A. aeolicus* possesses two putative cytochrome *c* oxidases (Figure 1) [5]. The terminal cytochrome oxidase produced in the growth conditions used here is the *ba*_3_-type enzyme. If the second uncharacterized cytochrome *c* oxidase is expressed in our cultures, it must be at concentrations an order of magnitude lower than that for *ba*_3_ oxidase. As we assumed that a very low oxygen concentration is available during the growth of *A. aeolicus* (probably a few µg/L, at least at the end of growth in our conditions), it seems logical to us that the *ba*_3_ terminal oxidase is predominantly expressed. Indeed, under low aeration conditions, the preferred oxygen reduction pathway in *T. thermophilus* is the *ba*_3_ branch and it has been proposed that the *ba*_3_ enzyme, with a high affinity to O_2_, is evolutionary selected to work under low oxygen tension [35]–[37]. It is also the case in Archaea [17], [21]. The aim is now to grow *A. aeolicus* with various controlled oxygen concentrations in the medium to investigate the presence of both cytochrome *c* oxidases in the membranes of the bacterium, as well as the one of the *bd*-type quinol oxidase.

## Materials and Methods

### Isolation of the *A. aeolicus* supercomplex

The supercomplex was purified as previously described [6]. Briefly, it can be extracted from *A. aeolicus* membranes with 1% (w/v) dodecyl-β-D-maltoside in 50 mM Tris-HCl (2-Amino-2-(hydroxymethyl)propane-1,3-diol-HCl) pH 7.6, 5% (v/v) glycerol, 750 mM aminocaproic acid at 37°C for 1 h. After membrane solubilization, the supercomplex is purified in three chromatographic steps.

### Denaturing gel electrophoresis

Purified supercomplex (70 µg) was loaded on a 10–20% denaturing gel (Tris-Tricine Precast gels from Bio-Rad), using a mini-protean III cell electrophoresis apparatus (Bio-Rad) as described in [38]. After migration, gels were stained with Coomassie Blue R-250.

### N-terminal sequence determination

The N-terminal amino acid sequence was determined after SDS gel electrophoresis and transfer of proteins onto polyvinylidene difluoride (PVDF) membrane for 45 min at a current intensity of 5 mA.cm^−2^ using a semi-dry electrophoretic transfer unit (Biometra). Sequence degradation was performed on an Applied Biosystems Procise 494 microsequencer equipped by a high-pressure liquid chromatography for quantitative determination of phenylthiohydantoin derivates.

### DNA sequencing

To identify a potential error of *A. aeolicus* genome sequencing, PCR was used to generate a 325 bp DNA fragment located between the *coxA_1_* and *coxB_2_* genes (positions 1541988 to 1542007 and 1542333 to 1542353) using the (5′-GTCGGGTACAATAGCATTTT-3′ and 5′-CGTGAATATGTCCACTACGGA-3′) oligonucleotide pairs. The DNA fragment was sequenced using the Sanger method (Beckman Coulter Genomics). The nucleotide sequence and annotation have been deposited to the European Nucleotide Archive under accession number FR846386.

### MALDI-TOF mass spectrometry

1 µL of a 22 µM solution of intact proteins was directly spotted onto a MALDI stainless steel target plate and an equal volume of a saturated solution of matrix sinapinic acid (40% CH_3_CN in water, 0.1% Trifluoroacetic acid (v/v)) was added (n = 3). Then, mixtures were allowed to dry at room temperature. Data acquisition was operated on MALDI-TOF mass spectrometers Ultraflex II and Microflex II from Bruker Daltonics using the Flex control software. The methods used was either the linear positive mode with a pulsed ion extraction for studying proteins in the range of 1–30 kDa and 10–80 kDa, either the reflectron positive mode for characterization of the subunit IIa in the 1–10 kDa range. External mass calibration was carried out on the Protein calibration standard I or Peptide calibration standard (Bruker Daltonics). In linear mode, mass spectra were treated by a Gaussian smoothing. MS/MS analysis on the ion precursor at m/z 5105.99 was performed in a lift mode on the MALDI-TOF/TOF Ultraflex II mass spectrometer.

### Sequence analysis

Sequence alignments were performed using Clustal W [39]. Transmembrane helices were predicted with the TMHMM Server v. 2.0 (http://www.cbs.dtu.dk/services/TMHMM-2.0/). Sequences were retrieved *via* the NCBI server (htpp://www.ncbi.nlm.nih.gov/). The theoretical molecular masses of the proteins were calculated with the *Compute pI/Mw* tool (ExPASy Proteomics Server, http://us.expasy.org/tools/pi_tool.html) or was given by NCBI.

For the analysis, oxidases described as belonging to the B family were used [12], [16], [17], [18]. We used also sequences of oxidases found with a BLAST search (http://www.ncbi.nlm.nih.gov/sutils/genom_table.cgi) using the subunit II of *A. aeolicus*. For these latter sequences, a multiple sequences alignment of all the subunits I from these enzymes indicated the presence of the four conserved residues proposed to be involved in the K channel of B-type oxidases (Tyr 226, Tyr 237, Ser 300, Thr 303, *A. aeolicus* numbering) [2]. When not annotated, genes encoding putative subunits IIa in micro-organisms were searched in the DNA genomic sequence, directly upstream subunit II gene, using *Translate* tool (ExPASy Proteomics Server, http://us.expasy.org/tools/dna.html) to detect the potential ORFs and TMHMM Server v. 2.0 to predict the transmembrane helices in the putative proteins. Potential small proteins were considered as putative subunits IIa only if they contain a transmembrane domain (otherwise they were not considered).

### Molecular modeling

Since there is no crystallographic structure determined for *A. aeolicus ba*_3_ cytochrome oxidase, a homology model was built using SWISS-MODEL protein modeling program (version 8.05) which is available from the ExPASy website [40], [41]. The modelization of the three subunits of the complex is based on the modified sequence of *A. aeolicus* genes cytochrome oxidase II (see section *A. aeolicus* genomic sequence analysis). The *T. thermophilus ba*_3_ cytochrome oxidase X-ray crystal structure (protein data bank entry 1XME) was used as a template, the sequences of *A. aeolicus ba*_3_ oxidase subunits being at least 60% similar to those of the enzyme from *T. thermophilus*. Hemes and copper ions from the crystal structure were included in the model. The procedure was carried out separately for subunit I, subunit II and subunit IIa.

*T. thermophilus* subunit I contains 13 transmembrane helices [7], [8]. Search for membrane-spanning helices using the TMHMM server in the *Aquifex* subunit I revealed only 12 transmembrane helices. As a consequence, the sequence of *Thermus* subunit I used for modeling did not contain the 46 C-terminal amino acids.

The homology model was checked for correct stereochemistry by performing a Ramachandran analysis. The final model has more than 95% of the residues in the allowed regions. The resulting theoretical model was displayed and analyzed with WebLab ViewerLite 5.0 software (Accelrys Inc.).

## Supporting Information

Figure S1 **Multiple sequence alignment of some bacterial and archaeal oxidase subunits II.** These proteins correspond to putative or characterized *ba*_3_ cytochrome *c* oxidases. Residues identical in all sequences are shown in red, strongly similar residues in green and weakly similar residues in blue. Grey shaded residues in the *Aquifex* sequence are involved in a putative transmembrane helix (TMHMM Server v. 2.0, Prediction of transmembrane helices in proteins). Boxed residues in the *Thermus* sequence form a transmembrane helix in the three-dimensional structure of the enzyme (PDB 1XME). The Histidine and Cysteine residues ligands of the Cu_A_ site are shown in bold (His 96, Cys 131, Cys 135 and His 139, *A. aeolicus* numbering). The alignment has been made with ClustalW. Complete name of organisms and accession numbers of sequences used to create the alignment are given in Table 1.
(TIF)

Figure S2 **Multiple sequence alignment of halocyanins and subunits IIa.** Residues identical in all sequences are shown in red, strongly similar residues in green and weakly similar residues in blue. The alignment has been made with ClustalW. Only the C-terminal part of the halocyanin sequences from *Halobacterium salinarum* R1 (YP_001689974) and *Haloarcula marismortui* ATCC 43049 (YP_135809) are shown. Full length sequences of subunits IIa from *Natrialba magadii* ATCC 43099 (YP_003481904), *Natronomonas pharaonis* (CAA71529), *Haloterrigena turkmenica* DSM 5511 (YP_003402036), *Haloferax volcanii* DS2 (YP_003535000), *Halorubrum lacusprofundi* ATCC 49239 (YP_002567056) and *Aquifex aeolicus* VF5 are shown.
(TIF)
