# Supplementary figures and images for: The Elusive Third Subunit IIa of the Bacterial B-Type Oxidases: The Enzyme from the Hyperthermophile Aquifex aeolicus
Source: PLoS One. 2011 Jun 30;6(6):e21616. doi: 10.1371/journal.pone.0021616 (PMC3128077; doi:10.1371/journal.pone.0021616)

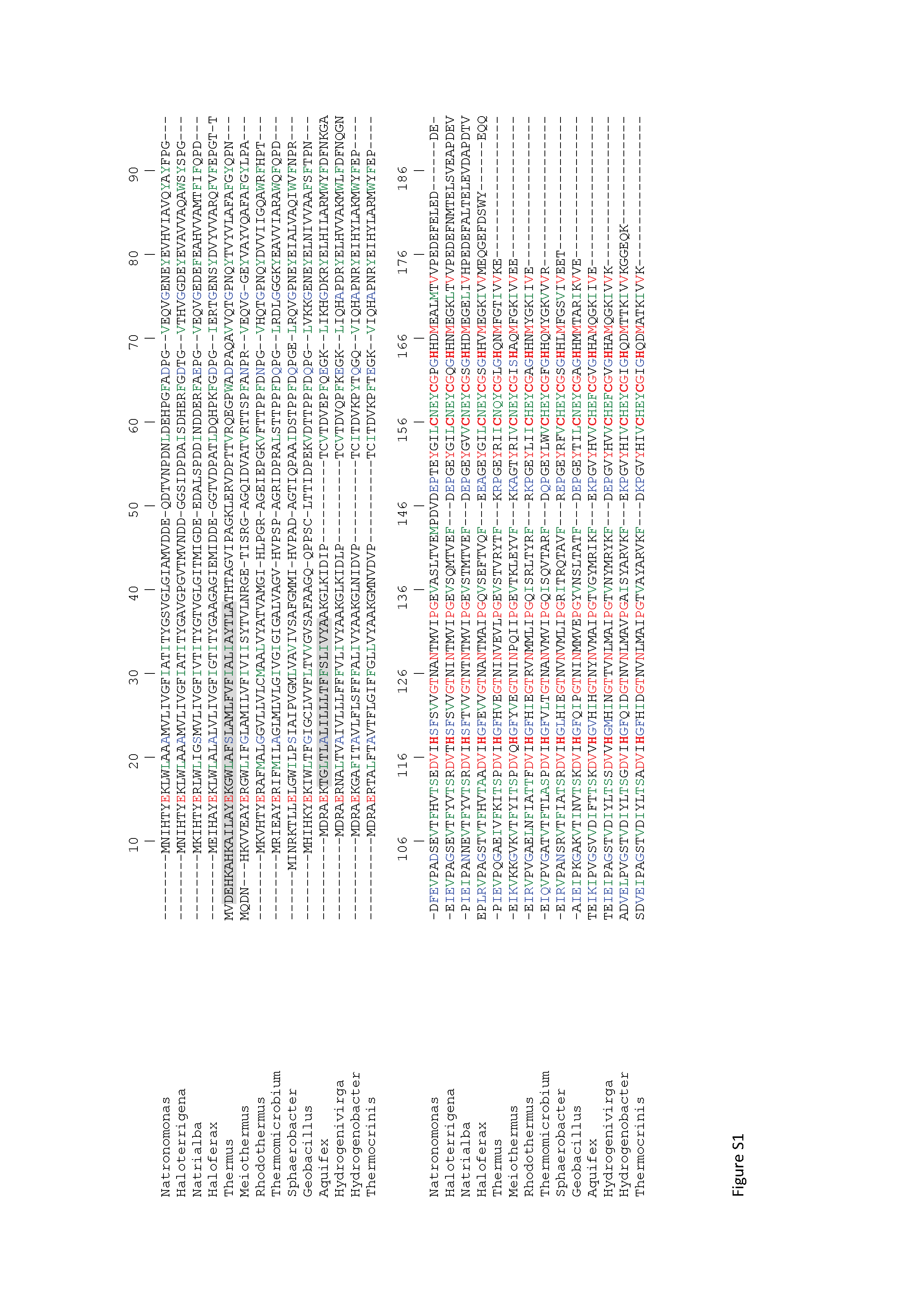

Supplement: Figure S1 — Multiple sequence alignment of some bacterial and archaeal oxidase subunits II. These proteins correspond to putative or characterized ba 3 cytochrome c oxidases. Residues identical in all sequences are shown in red, strongly similar residues in green and weakly similar residues in blue. Grey shaded residues in the Aquifex sequence are involved in a putative transmembrane helix (TMHMM Server v. 2.0, Prediction of transmembrane helices in proteins). Boxed residues in the Thermus sequence form a transmembrane helix in the three-dimensional structure of the enzyme (PDB 1XME). The Histidine and Cysteine residues ligands of the CuA site are shown in bold (His 96, Cys 131, Cys 135 and His 139, A. aeolicus numbering). The alignment has been made with ClustalW. Complete name of organisms and accession numbers of sequences used to create the alignment are given in Table 1. (TIF) [file pone.0021616.s001.tif]

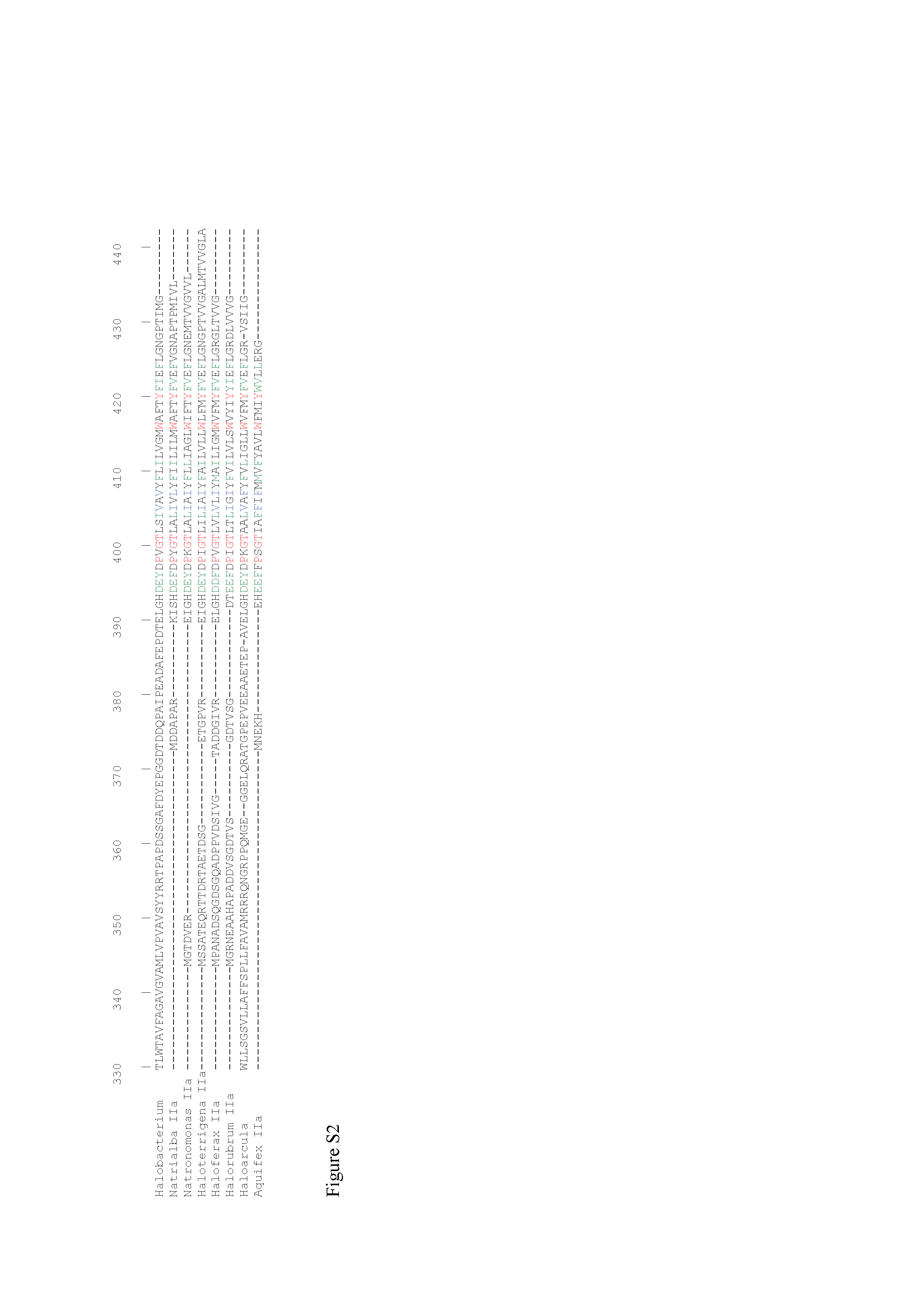

Supplement: Figure S2 — Multiple sequence alignment of halocyanins and subunits IIa. Residues identical in all sequences are shown in red, strongly similar residues in green and weakly similar residues in blue. The alignment has been made with ClustalW. Only the C-terminal part of the halocyanin sequences from Halobacterium salinarum R1 (YP_001689974) and Haloarcula marismortui ATCC 43049 (YP_135809) are shown. Full length sequences of subunits IIa from Natrialba magadii ATCC 43099 (YP_003481904), Natronomonas pharaonis (CAA71529), Haloterrigena turkmenica DSM 5511 (YP_003402036), Haloferax volcanii DS2 (YP_003535000), Halorubrum lacusprofundi ATCC 49239 (YP_002567056) and Aquifex aeolicus VF5 are shown. (TIF) [file pone.0021616.s002.tif]
